# Conservation and utilization of African *Oryza* genetic resources

**DOI:** 10.1186/1939-8433-6-29

**Published:** 2013-10-29

**Authors:** Peterson W Wambugu, Agnelo Furtado, Daniel LE Waters, Desterio O Nyamongo, Robert J Henry

**Affiliations:** Queensland Alliance for Agriculture and Food Innovation, The University of Queensland, Brisbane, St Lucia, Qld Australia; Southern Cross Plant Science, Southern Cross University, Lismore, NSW Australia; Kenya Agricultural Research Institute, Nairobi, Kenya

**Keywords:** Conservation, Germplasm, Genetic diversity, Germplasm utilization, Wild rice, Genomic sequences

## Abstract

Africa contains a huge diversity of both cultivated and wild rice species. The region has eight species representing six of the ten known genome types. Genetic resources of these species are conserved in various global germplasm repositories but they remain under collected and hence underrepresented in germplasm collections. Moreover, they are under characterized and therefore grossly underutilized. The lack of in situ conservation programs further exposes them to possible genetic erosion or extinction. In order to obtain maximum benefits from these resources, it is imperative that they are collected, efficiently conserved and optimally utilized. High throughput molecular approaches such as genome sequencing could be employed to more precisely study their genetic diversity and value and thereby enhance their use in rice improvement. *Oryza sativa* was the first crop plant to have its reference genome sequence released marking a major milestone that opened numerous opportunities for functional characterization of the entire rice genome. Studies have however demonstrated that one reference genome sequence is not enough to fully explore the genetic variation in the *Oryza* genus, hence the need to have reference sequences for other species in the genus. An overview of the state of conservation and utilization of African *Oryza* is hereby presented. Progress in the release of reference genome sequences for these species is also highlighted.

**Electronic supplementary material:**

The online version of this article (doi:10.1186/1939-8433-6-29) contains supplementary material, which is available to authorized users.

## Introduction

**Figure 1:**
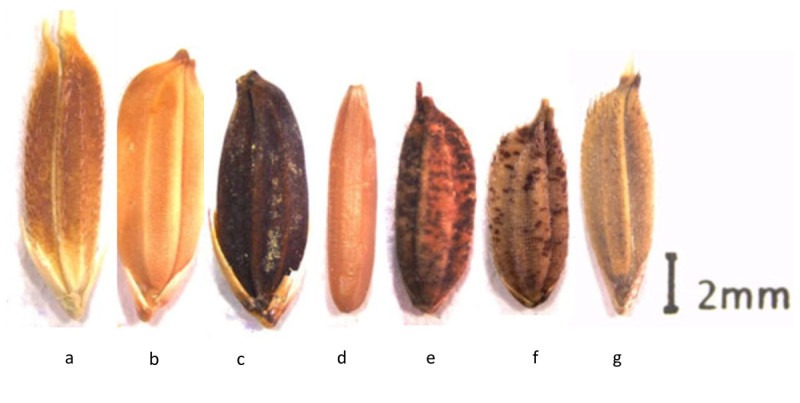
**Undehusked seeds of African** ***Oryza*** **species. (a)** *Oryza longistaminata* **(b)** *Oryza glaberrima* 1 **(c)** *Oryza glaberrima* 2 **(d)** *Oryza brachyantha* **(e)** *Oryza eichingeri* **(f)** *Oryza punctata* **(g)** *Oryza barthii.*

The *Oryza* genus has two cultivated species, *Oryza sativa* and *Oryza glaberrima*, and about 24 wild species (Lu and Jackson 2009; USDA-ARS 2013) representing ten rice genome types (Ge et al. 2001). With a total of eight species of both cultivated and wild rice species, representing six out of the ten known genome types (Table 1 and Figure 1), the African region is, arguably, one of the greatest sources of diversity in the rice gene pool. It is the only region where the two cultivated species co-exist by growing sympatrically. Unlike the Australian *Oryza* which has been genetically isolated from domesticated rice (Henry et al. 2009; Waters et al. 2012), African wild rice is found growing in contact with the cultivated taxa allowing extensive hybridization between cultivated and some of the wild rice species especially those in the AA genome (Lu and Snow 2005). Despite this possible hybridization, African wild rice species remain a largely untapped source of useful genetic and allelic diversity although they have made an important contribution in rice improvement (Brar and Khush 2002; Jena 2010; Khush et al. 1990).

**Table 1:** **African** ***Oryza*** **species, their chromosome numbers and genome types**

Species	2n	Genome type
*Oryza sativa* L.	24	AA
*Oryza longistaminata* A. Chev. et Roehr.	24	AA
*Oryza glaberrima* Steud*.*	24	AA
*Oryza barthii* A. Chev.	24	AA
*Oryza punctata* Kotschy ex Steud.	24	BB
*Oryza schweinfurthiana* Prod.^1^	48	BBCC
*Oryza eichingeri* A. Peter.	24	CC
*Oryza brachyantha* A. Chev. et Roehr.	24	FF

^1^*Oryza schweinfurthiana* Prod is also considered the tetraploid form of *Oryza punctata* Kotschy ex Steud. This paper makes no further reference to *Oryza schweinfurthiana* Prod. as there is negligible amount of data available on this species.

Despite the long history of rice research covering diverse areas and the demonstrated value of the African *Oryza* species, there is still little information on these species compared to others in the genus (Goicoechea 2009). World agriculture is facing unprecedented challenges largely attributed to climate change and the need to ensure proper conservation and sustainable utilization of genetic resources of crop wild relatives has never been more important (Brar 2004; FAO 2010). In order to ensure that maximum benefits accrue to humankind from these resources, it is imperative that they are collected, efficiently conserved and optimally utilized. The continued dramatic decrease in the costs of sequencing and genotyping has revolutionized genetic resources conservation and utilization and application of high throughput genomics is increasingly becoming common in genebank activities (McCouch et al. 2012). While information on the utilization and conservation of other wild species have been reviewed (e.g. Henry et al. 2009; Song et al. 2005), no such efforts have been made for the African *Oryza* species. This paper provides an overview of the status of conservation and utilization of African *Oryza* species and identifies gaps in knowledge and opportunities for further research.

## Review

### Phylogeny of African *Oryza*

Efficient conservation and utilization of the *Oryza* gene pool will require a clear understanding of the phylogeny and evolutionary relationships of the various species in addition to a reliable taxonomic and biosystematics framework. In the last two decades, efforts have been made in studying the phylogenetic relationships in the *Oryza* genus using a variety of approaches. These have mainly entailed the use of molecular markers such as Random Amplified Polymorphic DNA (RAPD; Bautista et al. 2001; Ishii et al. 1996; Ren et al. 2003), Simple Sequence Repeats (SSR; Ishii et al. 1996; Ren et al. 2003), Restriction Fragment Length Polymorphisms (RFLP; Bautista et al. 2001; Lu et al. 2002; Wang et al. 1992), Amplified Fragment Length Polymorphism (AFLP; Park et al. 2003a), Inter Sequence Simple Sequence Repeats (ISSR; Joshi et al. 2000), Short Interspersed Elements (SINEs) and Miniature Inverted-Repeat Transposable Element (MITE) insertions (Cheng et al. 2002; Iwamoto et al. 1999; Mochizuki et al. 1993). In addition, morphological and cytological studies have also been conducted (Lu et al. 2000). These studies have significantly increased our understanding of the evolutionary relationships in the *Oryza* but many questions remain unanswered (Tang et al. 2010; Zou et al. 2008). Resolving apparent phylogenetic incongruences in the *Oryza* genus must therefore remain a major focus of research for some African species.

**Figure 2:**
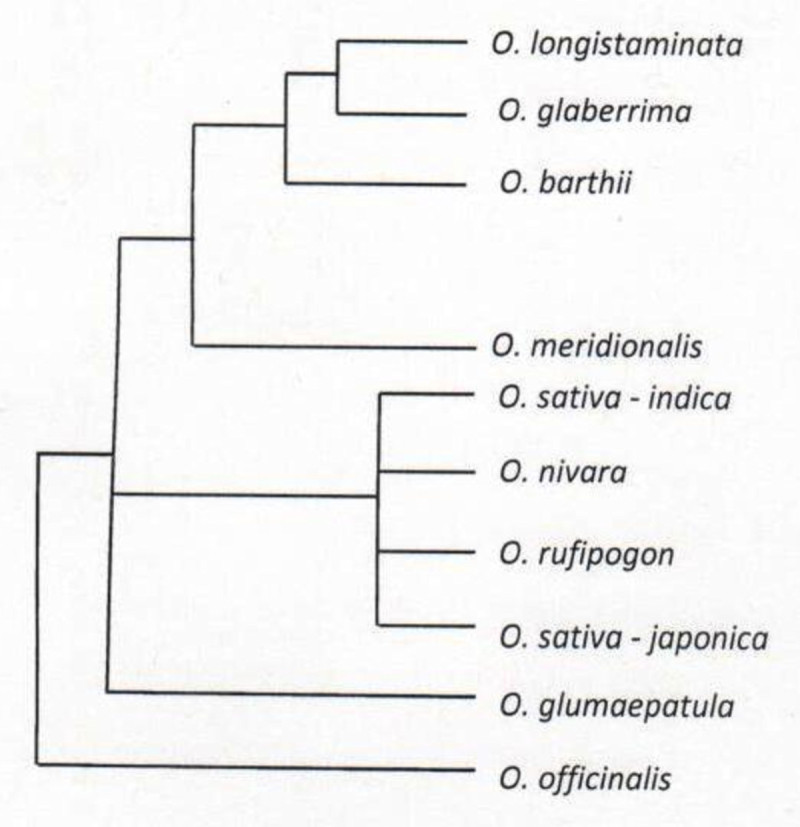
Consensus tree of the ***A*** **A genome rice species based on** ***trn*** **L-** ***trn*** **F sequences (Adapted from Duan et al.** 2007).

Among the African species, *Oryza brachyantha* has been the most affected by the conflicts resulting in several inconsistent phylogenetic placements depending on the phylogenetic approach used. For example, a phylogenetic study based on *Adh1*, *Adh2* and *matK* genes conducted by Ge et al. (1999) resulted in 3 different phylogenies all with inconsistent positioning of *Oryza brachyantha*. The *Adh2* phylogeny suggested a strong relationship between *O. brachyantha* and the diploid HH genome which was not supported by the *Adh1* phylogeny. The 3 phylogenies constructed by these authors depicted *O. meyeriana* and *O. granulata* (GG) as the most basal taxa, a finding that was inconsistent with the phylogenetic analysis by Mullins and Hilu (2002) where *O. brachyantha* appeared as the most basal lineage in the whole genus. Due to this discordance, Ge et al. (1999) recommended the use of additional genes in future phylogenetic studies. The most tenuous placement of *O. brachyantha* was by Wang et al. (1992) and Nishikawa et al. (2005), who, despite *O. brachyantha* being one of the most divergent species, placed it closest to the *Oryza sativa* complex. According to Goicoechea (2009), this seemingly controversial placement of *Oryza brachyantha* could be attributed to sequence conservation between AA and FF genomes in some of the loci under investigation. Just like *O. brachyantha* (FF), the AA genome species have also suffered from phylogenetic incongruence. For example, based on MITE-AFLP and RFLP data, Wang et al. (1992) and Park et al. (2003b) found *O. meridionalis* to be the most distinct species. This was contradicted by Ren et al. (2003), who, by conducting an SSR and RAPD analysis, found *Oryza longistaminata* to have undergone significant differentiation from the other AA genome species, a finding that was consistent with other previous studies (Cheng et al. 2002; Iwamoto et al. 1999). Figure 2 shows a consensus tree of the AA genome based on *trn* L-*trn* F sequences. Understanding the causes of these different conclusions may help to resolve them.

The differential results in the various phylogenetic analysis can be attributed to misidentification of accessions, gene choice, use of insufficient data, introgression and hybridization, rapid speciation as well as lineage sorting (Wang et al. 1992; Zhu and Ge 2005; Zou et al. 2008). Results from many studies provide evidence which shows that some of the previously unresolved phylogenetic relationships could be resolved by the use of more data, better selection of genes as well as better phylogenetic methods (e.g. Cranston et al. 2009; Zou et al. 2008). The importance of proper choice and use of a sufficient number of unlinked genes in resolving phylogenetic studies was demonstrated by a recent study that used a total of 142 single copy genes to resolve the phylogeny of all the diploid genomes of *Oryza* (Zou et al. 2008). Moreover, the use of chloroplast DNA as opposed to nuclear data has been suggested as one way to reduce incongruence in phylogenetic relationships (Takahashi et al. 2008; Waters et al. 2012). Chloroplast DNA is haploid, non-recombinant and is generally maternally inherited thus making it a useful tool for molecular systematics (Small et al. 2004; Waters et al. 2012). A recent study of the *Oryza* phylogeny generated a better resolved phylogeny based on 20 chloroplast fragments (Tang et al. 2010). However, the potential of chloroplast DNA in resolving phylogenetic relationships as highlighted by these studies is contradicted by other studies where the use of chloroplast fragments has resulted in inconsistent phylogenies or lack of sufficient resolution (Guo and Ge 2005). It would appear that this apparent phylogenetic inconsistency based on chloroplast data may be due to the number of genes or DNA fragments studied. As with nuclear data, increasing the number of chloroplast loci included may increase resolution. Indeed, Waters et al. (2012) reported that the use of whole chloroplast genome sequences has more power in phylogenetic resolution. However, this molecular tool has not been fully exploited in reconstructing phylogeny of the *Oryza*. On the other hand, with the continued advances in bioinformatics and increased computation capacity, it is expected that the use of whole genome sequences in resolving phylogenetic relationships will soon become feasible, thus increasing resolution. Better information on the phylogenetic relationships in the *Oryza* genus will facilitate more efficient conservation and utilization in rice crop improvement programs.

### Germplasm conservation of African *Oryza*

Plant genetic resources underpin world agriculture and their conservation is therefore imperative if food security and sustainable development is to be assured. To define the current status of African *Oryza* germplasm conservation, we review approaches that are currently being used in the conservation of wild rice germplasm ex situ and in situ*.* Details of available germplasm collections and a brief analysis of collection gaps in ex situ conservation, is presented.

### Ex situ conservation

#### Collection and conservation

Ex situ conservation is the most common germplasm conservation method and can take the form of seed banks, field genebanks or botanic gardens. DNA storage in DNA banks represents another option for the conservation of genetic materials (Henry et al. 2009). Collection of wild rice relatives started in the 1950s reaching a peak in the 1980s and 1990s when the International Board for Plant Genetic Resources (IBPGR, formerly IPGRI and now Bioversity International) was formed. IBPGR, in collaboration with both international and national partners funded and spearheaded most of the African wild rice germplasm collection efforts. This was due to the realization that these valuable resources faced eminent threat of genetic erosion due to both environmental and socio-economic factors. The collected materials were deposited in national genebanks with duplicates being conserved in international genebanks especially the International Rice Research Institute (IRRI;IBPGR 1984). Currently, IRRI holds the largest collection of African *Oryza* totaling to about 3601 accessions (Table 2). Other collections of African *Oryza* can be found in Australian Tropical Grains Germplasm Centre; International Network for the Genetic Evaluation of Rice in Africa (INGER-Africa); Bangladesh Rice Research Institute, Dhaka; SADC Plant Genetic Resources Centre (SPGRC), Zambia and China National Rice Research Institute (CNRRI) (Agnoun et al. 2012; Hay et al. 2013). Duplicate samples of African *Oryza* collections are shared between IRRI and the Africa Rice Center (previously known as WARDA) in Benin, Africa (Brar and Singh 2011). As in other species, the level of unintended duplication is high in rice thus escalating the costs of conservation. Many strategies and methods have been used to identify duplicates but most of these have been unsuccessful. However, high throughput genotyping and sequencing has the capacity to more precisely identify duplicate samples in the future as well as enable a more accurate analysis of available collection gaps.

**Table 2:** **Regional, International and genebanks outside Africa holding African** ***Oryza*** **germplasm**

Species	IRRI, Philippines^1^	Africa Rice Centre^1^	Australian Plant DNA Bank^2^	Millennium Seed Bank Project^1^	USDA^3^	ILRI, Ethiopia^1^	National Institute of Genetics^4^	SPGRC
*O. glaberrima*	2828	3796	7	1	476	1		
*O. barthii*	331	114	5	5	15	1	386	2
*O. longistaminata*	285		2	3	9	5	149	54
*O. eichingeri*	37		6	0	0	0	11	
*O. punctata*	89		0	1	14	2	18	
*O. brachyantha*	31		0	1	6	1	16	2

^1^http://www.genesys-pgr.org/.

^2^http://collections.ala.org.au/public/show/co133.

^3^ USDA-ARS (2013).

^4^ Nonomura et al. (2010).

#### Collection gap analysis

The size of collections conserved in the genebanks (Table 2) suggests that maximum genetic diversity has been captured. However, effective management of these genebanks and conservation of their resources would benefit from detailed gap analysis which would help guide future conservation priorities. Such an analysis would help to determine if all the genetic diversity found in a taxa is represented in in situ as well as ex situ conservation facilities (Maxted et al. 2008). While the combined use of molecular data, eco-geographic surveys and more accurate geographic referencing is vital in identifying gaps and redundancies in existing collections (FAO 2010), very little of this has been done for African wild *Oryza* held in many national collections. With the rapidly changing nature of African agriculture and in the face of increasing documented threats to biodiversity in the region (Khumalo et al. 2012; Wambugu and Muthamia 2009), there is need to use these tools to more precisely and systematically understand and document this genetic diversity as well as the status of its conservation. A comparative study of the diversity conserved ex situ with that found in situ would be particularly useful as it would help identify some possible collection gaps. There however seems to be no published work on such comparative studies for African *Oryza*. The success of a collection gap analysis is partially dependent on the quality and integrity of the available data (Ramirez-Villegas et al. 2010) and currently, paucity in the necessary data remains a major constraint in undertaking a comprehensive collection gap analysis. Available data indicates that globally, there are no collection deficiencies at the taxa level as all the taxa are represented in ex situ facilities. However, an analysis of germplasm collection data (Additional file 1: Tables S1 and S2) and herbarium specimens (Additional file 1: Tables S3 and S4) for individual in-country collections reveals some significant gaps in taxa coverage. For example, in Kenya, where the authors had access to comprehensive and reliable data, there is a clear taxonomic gap in the collection of *Oryza eichingeri*. A herbarium specimen was collected and deposited at Missouri Botanic Garden (http://www.tropicos.org/Specimen/3179202) but no germplasm samples of the same have ever been collected.

In contrast to other species such as *Phaseolus sp*. (Ramirez-Villegas et al. 2010), the number of herbarium specimens in *Oryza* is higher than conserved germplasm accessions, a possible indicator of underrepresentation in ex situ conservation facilities. This observation is reinforced by a further numerical assessment of individual in-country germplasm collections (Additional file 1: Tables S1 and S2) which indicates that some of the wild species especially *Oryza eichingeri* and *Oryza brachyantha* have very few or no accessions in both national and international genebanks. A similar observation of under representation of *Oryza* wild species in ex situ conservation facilities was made by Maxted and Kell (2009). These two species had the lowest sampling representative score (SRS), of 5.9 and 5.6 respectively, with *O. glaberrima* having the highest followed by *Oryza longistaminata*. Sampling representative score (SRS) is an indicator of the adequacy of germplasm holdings for a particular taxon, based on available herbarium specimens and germplasm collections (Ramirez-Villegas et al. 2010). While acknowledging that the number of accessions may not always give an accurate reflection of the available diversity (FAO 2010), this data indicates some in-country collections with clearly evident collection gaps that may need to be filled. This finding aligns well with expert opinions on the existence of gaps in ex situ collection. For example, Hay et al. (2013) noted that there were indications of collection gaps of wild rice species in areas outside Asia such as Africa. Similarly, Ngwediagi et al. (2009) reported wild rice collection gaps in Tanzania. Using molecular approaches, efforts should be made to ensure that any targeted collection efforts arising from these collection gap analysis and recommendations, should only be undertaken if it has been established that it will result in new genetic or allelic diversity. The planned release of reference genome sequences of African *Oryza* is expected to provide a basis on which to assess available genetic diversity using high throughput genomic approaches. As observed by McCouch et al. (2012), due to the continued dramatic decrease in the costs of sequencing and concomitant increase in efficiency, it is currently cheaper to undertake low coverage sequencing of an accession than it is to add an accession of cultivated rice to a germplasm collection. Consequently, it is expected that in future, genotyping using molecular markers or by sequencing will become a routine activity before an accession is banked to ensure that only those with novel alleles or allele combinations are added to the collection. In addition to these ex situ collections, the importance of putting in place extra safeguards to genetic resources, by establishing and maintaining in situ collections is now universally accepted.

### In situ conservation

**Figure 3:**
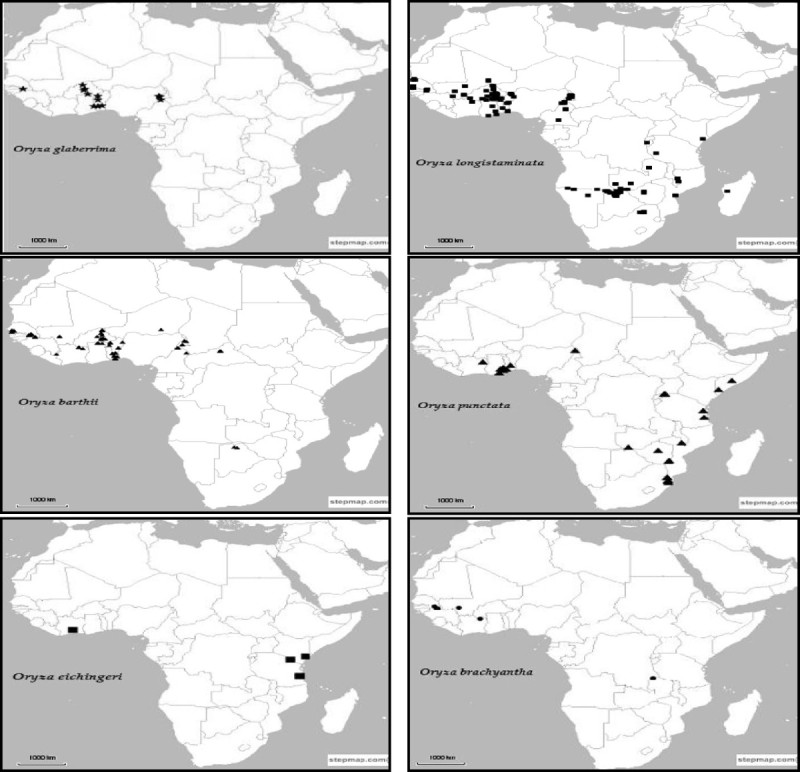
**Distribution of** ***Oryza*** **species in Africa.** Species distribution has been mapped based on records of herbarium specimens which have been preserved in various herbaria globally.

In situ conservation of wild species has for decades now been undertaken to complement ex situ conservation and is known to have particular advantages such as allowing the natural process of evolution to continue. However, despite the demonstrated importance of in situ conservation and several warnings on the alarming rates at which genetic diversity of rice wild relatives is being lost (Song et al. 2005; Vaughan and Chang 1992), there is no documented evidence of any targeted in situ conservation programs for African wild rice species (Brink and Belay 2006; Maxted and Kell 2009). This is in contrast to other rice species where several studies have indicated the presence of such programs (Song et al. 2005; Xie et al. 2010). The need to put in place a robust, complementary in situ conservation program for the African rice gene pool cannot be overemphasized and there is great agreement in the literature on the importance of such a program. Vaughan and Chang (1992) noted that ex situ conservation of wild species that exhibit great heterogeneity in their genetic structure is not only expensive but also time consuming, lending support to the need for in situ conservation. Maxted and Kell (2009) noted that in order to establish in situ genetic reserves, detailed genetic studies of wild rice species are vital as they help in identifying priority locations for in situ conservation. Such knowledge, supplemented with information on herbarium specimens (Additional file: 1: Table S3 and S4) and species distribution (Figure 3) will be important in supporting conservation and sustainable utilization management decisions. Detailed genetic diversity studies on the naturally occurring variation of African *Oryza* are however limited, thus constituting a major information gap that greatly hampers establishment of these reserves. Currently, while populations of African wild rice may be found occurring in nationally designated protected areas, these are just an indirect effect of the establishment of these areas and such populations benefit from no active management. In Tanzania, for example, Vaughan and Chang (1992) reported the occurrence of *O. barthii* and *O. punctata* in Ruaha National Park which is a protected habitat. Outside the protected areas, wild rice species may also be found in cultivated farmers’ fields, field edges, pasturelands, orchards, recreation parks and roads.

**Figure 4:**
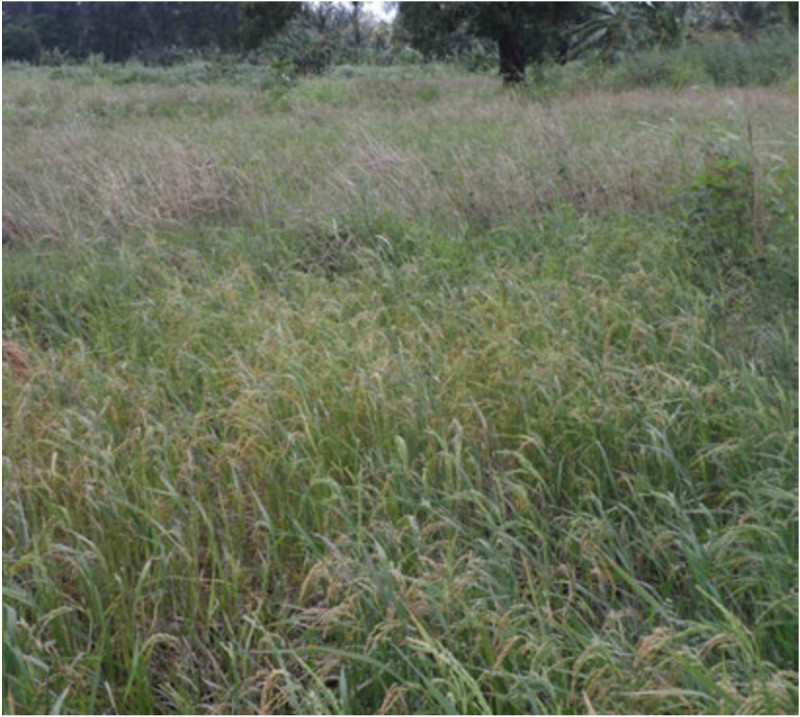
**Rice field in coastal Kenya planted with** ***Oryza sativa*** **landrace with patches of** ***Oryza punctata*** **.** The sympatric occurrence of cultivated and wild species may lead to gene flow thus affecting the genetic integrity of these species. It could also lead to extinction of less adapted genotypes.

Whether in protected or in non-protected areas, *Oryza* populations are at great risk of extinction due to threats of climate change, overgrazing, flooding, habitat change, invasive alien species and pollution. Climate change probably constitutes the greatest threat, with reports indicating that Africa, especially sub-Saharan Africa which holds considerable diversity of these species, will be adversely affected by climate variability. The challenge of climate change is expected to be serious particularly for wild species and urgent efforts are therefore needed to secure their genetic resources as the likelihood for extinction of narrowly adapted and endemic species is high (Jarvis et al. 2009). However, even in the face of threats posed by climate change, wild rice species are still expected to provide the basis for adapting agriculture to climate change in future. Those naturally occurring in situ populations that have the capacity to withstand the harsh effects of climate variability will have the potential to contribute valuable new traits for rice improvement (FAO 2010; Pettersson et al. 2009). In addition to these threats, an often neglected challenge facing in situ conservation is that of gene flow between cultivated and wild rice species and its impact on genetic diversity and integrity (Figure 4). While it is generally acknowledged that the transfer of genes from wild to cultivated taxa may have important and beneficial consequences, gene flow in the reverse direction (i.e. from the crop to the wild) may lead to deleterious changes in genetic diversity or even result in extinction of small populations (Ellstrand et al. 1999). It would appear therefore that establishment of genetically isolated reserves is the most viable and effective strategy to conserve and at the same time reduce genetic admixture and its associated consequences. However, such a recommendation needs to be based on comprehensive gene flow studies that allow assessment of the genetic integrity of the populations involved. Such studies have lately been undertaken using high resolution genotyping tools such as Single Nucleotide Polymorphisms (SNPs) and SNP haplotypes as well as sequencing technologies.

### On farm conservation

**Figure 5:**
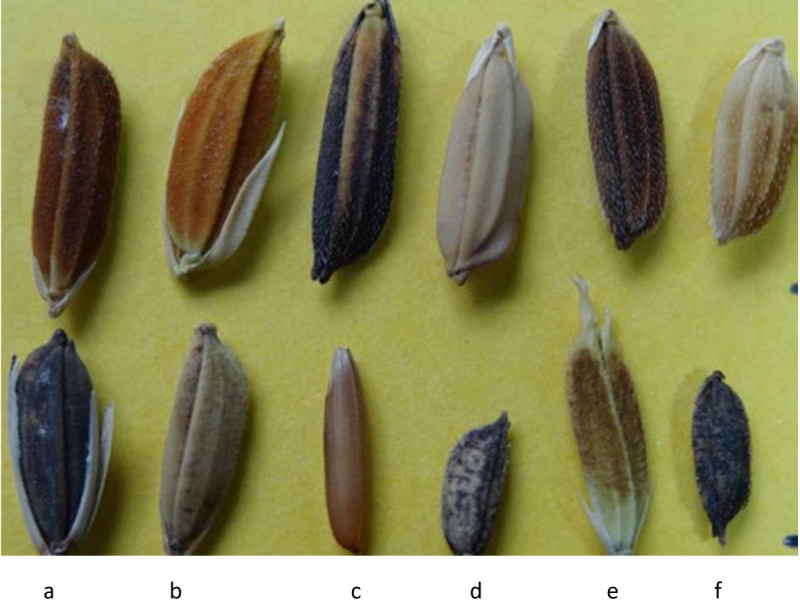
**Phenotypic diversity of both cultivated and wild** ***Oryza*** **genetic resources: Upper row,** ***Oryza sativa*** **landraces; Lower row, (a)** ***Oryza glaberrima,*** **(b, c, d, e, f) African wild** ***Oryza*** **species.**

During the course of farming, farmers have been known to maintain important genetic diversity of *Oryza sativa* and *Oryza glaberrima* within the farming system (Figure 5). This system of conservation, often referred to as on-farm conservation, is usually characterized by local/farmer seed production with little or no acquisition of certified seeds from the formal seed sector. Additionally, the system relies heavily on the use of diverse traditional varieties which usually have high levels of genetic diversity as compared to improved varieties. A study conducted in West Africa shows that about 70% of the farmers grow their traditional rice varieties (Mohapatra 2007). According to a National Research Council report, most of the farmers in this region have reported deliberate mixing of both the cultivated Asian and African rice in their farms so as to foster genetic diversity which occurs as a result of introgression. This has resulted in new types of landraces which, judging by ligule form, grain shape, and panicle type, are intermediate between the cultivated Asian and African rice (National Research Council 1996). These resultant landraces may be low yielding but are able to cope up with many biotic and abiotic stresses due to their heterogeneity and are therefore likely to lead to yield stability. Studies have shown that farmers prefer yield stability to maximum obtainable yields in line with their strategy of risk avoidance (Almekinders and Louwaars 1999; Wambugu et al. 2012). This observation is confirmed by reports that farmers in some parts of West Africa such as the *Banfora* area in Burkina Faso are abandoning improved rice varieties in favour of the low yielding but highly adaptable *Oryza glaberrima* varieties (Futakuchi et al. 2012). Barry et al. (2007) observed that the replacement of rice landraces by improved varieties in Africa is less advanced than in Asia. It is therefore important that these landraces are conserved on-farm as well as ex situ and genetic studies undertaken on them as they might harbour useful traits that may find utility in rice improvement.

### Utility of African *Oryza*: achievements and challenges

As already highlighted, a lot of efforts have been made in the collection and conservation of huge collections of African *Oryza* which are currently conserved in various facilities (Table 2), for the benefit of present and future generations. In order to derive maximum benefits from these resources, it is imperative that efforts are made to ensure their optimal utilization in research, crop improvement as well as direct use. Promoting the sustainable use of these plant resources requires an understanding of their value. This section therefore reviews some of the potentially desirable traits they possess as well as some of the progress achieved in incorporating them into commercial varieties.

### Genetic potential of African *Oryza*

African cultivated and wild rice species are known to possess enormous genetic diversity (Figure 5) which is of immense genetic value especially on resistance to biotic and abiotic stresses and therefore remain a vital raw material in rice improvement programs (Table 3). Efforts to use these resources have led to significant success in the transfer of useful traits into cultivated rice (Brar and Khush 2002). Hajjar and Hodgkin (2007) indicated that to date, a total of 12 traits in cultivated rice have been improved through the use of wild rice gene pool. One of the most significant and successful uses of wild genes in rice improvement is the transfer of the *Xa-21* gene conferring resistance against bacterial blight resistance which was successfully introgressed from *O. longistaminata* into *Oryza sativa* (Khush et al. 1990)*.* Several improved varieties carrying the *Xa-21* gene have subsequently been released in different countries.

**Table 3:** **Useful traits found in African** ***Oryza*** **species**

Species	Trait	Reference
*O. longistaminata*	Resistance to bacterial blight, nematodes, drought avoidance; rhizomatousness	(Brar and Khush 2002; Hu et al. 2011; Jena 2010; Khush et al. 1990; Yang et al. 2010)
*O. brachyantha*	Resistance to bacterial blight, yellow stem borer, leaf-folder, whorl maggot, tolerance to laterite soil	(Brar and Khush 2002; Ram et al. 2010; Yamakawa et al. 2008)
*O. glaberrima*	Resistance to drought , iron toxicity, nematodes; weed competitiveness; high adaptability to acidic soils showing low levels of phosphorus availability; cultigen; tolerance to waterlogging; crude protein content; cultigen; African gall Midge; stem borers; Rice yellow mottle virus; resistant to nematodes	(Brar 2004; Brar and Khush 1997,2002; Dingkuhn et al. 1998; Futakuchi et al. 2001; Jones et al. 1997; Li et al. 2004; Ndjiondjop et al. 1999; Nwilene et al. 2002; Plowright et al. 1999; Sauphanor 1985)
*O. barthii*	Resistance to green leaf hopper, bacterial blight, drought avoidance	(Brar and Khush 2002)
*O. punctata*	Resistance to brown plant hopper, zigzag leafhopper	(Brar and Khush 2002; Jena 2010)
*O. eichingeri*	Resistance to brown plant hopper, white-backed plant hopper, green leaf hopper	(Brar and Khush 2002; Yan et al. 1997)

Breeding for higher yields and yield stability remains the major objective of most rice breeding programs worldwide. Though wild rice species are phenotypically inferior and have predominantly been used as a source of genes for resistance to pests and diseases (Hodgkin et al. 2007), they also possess the genetic value necessary to improve the yield potential of cultivated rice (Xiao et al. 1998). However, as noted by Hajjar and Hodgkin (2007), the contribution of wild species in improving yields has been limited or almost non-existent, not only in rice but also in all other economically important crops. In a review of the use of wild relatives in crop improvement in 16 major economically important crops, these authors reported only one case of a released variety bred by incorporating yield enhancing genes from a wild rice species. The rice cultivar, NSICRc112, developed from a cross between *O. longistaminata* and *O. sativa* was released in the Philippines in 2002 and is known to be high yielding (Brar 2004). Similar to the case of yield improving genes, wild species have not contributed any genes to enhance drought tolerance in rice. This apparent lack of contribution of genes to improve quantitatively inherited traits such as yield can be attributed to the fact that it is difficult to phenotypically identify these superior traits in wild species (Bimpong et al. 2011). There is therefore need for more focus to be put on the use of molecular approaches in the identification of yield and drought related Quantitative Trait Loci (QTL) among other quantitative traits. In addition to breeding for stresses and yields, considerable attention has also been given to breeding for rice quality.

Rice quality is an important trait that largely determines its demand both at a household level as well as in international markets. Breeding for rice grain quality is therefore increasingly becoming a priority in breeding programs especially in developed countries. However, as noted by Henry et al. (2009), rice wild relatives have not yet contributed genes to improve the quality of rice. Both wild and cultivated African species however seem to have potential to contribute genes that will improve rice grain quality as well as acting as alternative food sources. In the case of African wild species, some species namely *O. longistaminata*, *O. punctata* and *O. barthii,* are consumed as whole grains in times of food scarcity thereby acting as a safety net for poor communities (Brink and Belay 2006). While it would therefore appear reasonable to suggest that these wild species have capacity for acting as new food crops, their nutritional properties are yet to be studied. Rice is mainly composed of starch and hence an analysis of the starch characteristics of these species and the diversity of genes encoding for these starches would be particularly important but has not been explored in African *Oryza* species. Starch biosynthesis genes have been studied extensively in cultivated rice resulting in identification of huge numbers of Single Nucleotide Polymorphisms (SNPs) in these genes, that have the potential to be used as molecular markers (e.g. Chen et al. 2008; Kharabian-Masouleh et al. 2011; Kharabian-Masouleh et al. 2012; Larkin and Park 2003; Umemoto et al. 2004; Umemoto et al. 2008). Kasem et al. (2012) identified a total of 251 SNPs in the exons of three starch genes namely *Grain Bound Starch Synthase I (GBSSI), Starch Branching Enzyme IIb (SBEIIb)* and *Starch Synthase IIa (SSIIa).* This study however included only Australian and Asian wild rice species leaving out African species. Grain morphology is another important quality trait that has an impact on the utilization of rice grains. In a study of grain morphology of a number of Australian wild rice species, Kasem et al. (2010) found a wide variation in grain morphological characteristics, specifically size and shape, but all of which were within standards acceptable to breeders. This study highlighted the potential utility of these wild species as whole grain foods as well as novel sources of important diversity for use in improving both *O. sativa* and *O. glaberrima*.

Despite being a cultivated species, *O. glaberrima* is arguably the most genetically promising of all African *Oryza*, possessing a rich repertoire of favourable genes and alleles that have the potential of improving a diverse range of agronomically important traits in rice (Table 3). Recent studies of a QTL analysis of an *O. sativa* and *O. glaberrima* cross indicated that *O. glaberrima* contains useful QTL alleles that are likely to significantly enhance traits including yield and other yield components particularly under conditions of drought stress. Some of these alleles are potentially novel and have been shown to be stable across genetic backgrounds (Bimpong et al. 2011). This cultivated African species also seems to be a new source of valuable genes for improvement of specific rice qualities such as eating, cooking and milling properties of rice grain, qualities that are valued in some rice export markets (Aluko et al. 2004). Furthermore, QTL analysis of progenies derived from interspecific crosses between *Oryza sativa* and *Oryza glaberrima* has revealed some loci in *O. glaberrima* that could enhance crude protein content and improve grain shape and appearance (Aluko et al. 2004; Li et al. 2004). Increased protein content is particularly desirable for poor regions such as Asia and Africa where malnutrition is widespread. Interspecific crosses between *O. sativa* and *O. glaberrima* resulted in interspecific hybrids trademarked as New Rice for Africa (NERICA) (Futakuchi and Sié 2009; Jones et al. 1997). NERICA varieties combine some of the stress tolerance traits of *O. glaberrima* with the high yielding potential of *O. sativa* (Jones et al. 1997).

Despite its great potential for utility in rice improvement programs, *O. glaberrima* has some negative traits namely poor yield, seeds that split and shatter easily, a notorious difficulty of milling and plants that lodge easily (Linares 2002; National Research Council 1996). Moreover, the brown or red pericarp is also not appealing to most consumers and in most cases the rice has to be polished to remove it (Teeken et al. 2012). It is perhaps due to these reasons that it is rapidly being displaced in West Africa in favour of Asian rice (Jones et al. 1997; Linares 2002) and is currently almost becoming extinct (Mohapatra 2010). While this calls for urgent breeding efforts, there is also need to give more attention to the conservation of this germplasm before it gets lost. Moreover, the conserved germplasm needs to be properly characterized in order to unravel its genetic architecture.

### Characterization of African *Oryza*

Successful utilization of genetic resources especially of wild species in breeding programs as well as in other research primarily requires some understanding of their phenotypic and genotypic characteristics as this knowledge increases the potential value of these resources. Moreover, this knowledge is important in making important germplasm conservation management decisions. Using AFLPs, Kiambi et al. (2005) studied the genetic diversity and population structure of 48 *O. longistaminata* populations obtained from 8 Eastern and Southern Africa countries. The study revealed higher levels of genetic diversity as compared to some other species in the *Oryza* genus such as *O. glumaepatula*. This diversity was found to be more within than between populations and also to be more in populations within countries than among countries. Similar results were found in a recent study involving 320 accessions of *O. longistaminata* obtained from 8 populations in Ethiopia (Melaku et al. 2013). This study found more within than between population diversity but the level of diversity was higher than the one detected in the study by Kiambi et al. (2005). In another study on genetic diversity and domestication history, Li et al. (2011) found 70% less diversity in *O. glaberrima* as compared to *O. barthii*, its wild progenitor, an indication of severe domestication bottleneck. These and other similar studies have been valuable not only in ensuring effective germplasm utilization but also in setting conservation priorities and defining sampling strategies. It is on the basis of outputs from such genetic diversity and evaluation studies that most of the *Oryza* genomic resources have been developed.

Currently, the *Oryza* research community has access to numerous genomic resources among them a reference sequence, advanced mapping populations, transcriptome data as well as physical and genetic maps. *Oryza sativa* was the first crop plant to have its reference genome sequence released (International Rice Genome Sequencing Project 2005) marking a major milestone that opened numerous opportunities for functional characterization of the entire rice genome. Studies have however demonstrated that one reference genome sequence is not enough to fully explore the genetic variation in the *Oryza* genus (Goicoechea et al. 2010). Consequently, efforts to develop reference genome sequences for some other selected species in the *Oryza* genus have been on-going under the auspices of the International *Oryza* Map Alignment Project (IOMAP). Through this initiative, physical maps have been developed for some selected species among them some African rices namely *O. glaberrima*, *O. punctata* and *O. brachyantha* (Kim et al. 2008). Genome sequences of *O. glaberrima*, *O. barthii*, *O. punctata and O. longistaminata* are available in Genbank though they are yet to be published (Table 4) (Jacquemin et al. 2013). Recently, *O. brachyantha* became the first African species to have its reference genome sequence published (Chen et al. 2013). With the expected release of reference genome sequences for the other wild species and with the continued decline in next generation sequences, it is anticipated that it will become increasingly feasible to study intraspecific genetic diversity by comparing individual genome sequences with reference sequence. Moreover, the release of these reference genome sequences will usher in a new platform that will give impetus to re-sequencing efforts which will in turn yield valuable information on SNPs, insertions, deletions, and other mutations as well as other structural and functional variations.

**Table 4:** **Sequencing status of African** ***Oryza*** **genomes**

Species	Genome size	Sequencing method	Status
*O. sativa* ssp. *indica*	≈400 Mb	WGS	2002 (Draft)
*O. sativa* ssp. *japonica*	≈400 Mb	CBC/PM	2004 (RefSeq)
*O. glaberrima*	≈354 Mb	BP	2010 (RefSeq)
*O. barthii*	≈411 Mb	WGS/PM	2012 (RefSeq)
*O. brachyantha*	≈260 Mb	WGS/PM	2011 (RefSeq)
*O. longistaminata*	≈352 Mb	WGS	2011 (Draft)
*O. punctata*	≈423 Mb	BP/WGS/PM	2012 (RefSeq)
*O. eichingeri*	≈650 Mb	WGS	Sequencing in progress

Key: *WGS* whole genome shotgun, *CBC* clone by clone, *BP* BAC Pool, *PM* physical map integration.

Modified from Jacquemin et al. (2013).

Numerous studies have been undertaken aimed at genome wide-discovery of DNA polymorphisms such as SNPs and InDels in the *Oryza* genus. These studies have led to discovery of huge numbers of DNA polymorphisms (Feltus et al. 2004; Shen et al. 2004; Subbaiyan et al. 2012; Xu et al. 2012). However, despite discovery of these DNA polymorphisms, a significant gap exists in linking them to phenotypic traits so that they can be of more value in crop improvement as well as in genetic diversity analysis. Such information, according to Tung et al. (2010), will lead to more insights on the value of naturally occurring variation and hence lead to better management and utilization of the biodiversity that is conserved in various global germplasm repositories. The integration of this information with genebank accession level information and data is expected to increase the value of genebank collections and thereby boost their utilization in rice improvement as well as other areas of plant science. Among the greatest challenges that genebanks currently face in this endeavor is the inadequacy in bioinformatics skills and computing capacity to handle the huge amount of genomic data that is being generated through sequencing and genotyping. Attempts towards proper integration of genomic and phenotypic data, that allow meaningful downstream analysis first calls for concerted efforts in compiling and publicly sharing genebank’s accession level information on characterization and evaluation. The lack of this information arguably presents the greatest obstacle to the effective use of conserved genetic resources (FAO 2010; Khoury et al. 2010; Wambugu et al. 2010). Without evaluation data, for example, it is not possible to link sequence polymorphisms with phenotypic performance. African *Oryza* thus remains greatly under characterized and hence grossly underutilized in rice breeding programs and its imperative that more focus is put in this important area. With the rapidly decreasing costs of next generation sequencing, there is need to start positioning genebanks for the ultra-high throughput genomic era which promises to revolutionize germplasm conservation and utilization as well as all associated activities.

## Conclusion

Undoubtedly, African *Oryza* genetic resources have numerous traits of potential value in the improvement of cultivated rice. The under representation of these resources in global germplasm repositories and the threats they face especially in the wild, call for concerted efforts, nationally, regionally and internationally, to ensure they are collected and properly conserved. The current advances and cost reduction in next generation sequencing, promises to revolutionize the conservation and utilization of these resources. These sequencing technologies should therefore be fully deployed in the characterization and utilization of these resources for the benefit of human kind.

## Electronic supplementary material

Additional file 1:**Table S1.** Germplasm collections held in various national genebanks. **Table S2.** Germplasm collected from selected countries and currently held in international genebanks. **Table S3.** Number of herbarium specimens of African *Oryza* species held in various herbaria globally. **Table S4.** Number of herbarium specimens of African *Oryza* collected from different African countries. (DOCX 30 KB)

Below are the links to the authors’ original submitted files for images.

Authors’ original file for figure 1

Authors’ original file for figure 2

Authors’ original file for figure 3

Authors’ original file for figure 4

Authors’ original file for figure 5

Authors’ original file for figure 6

## Abbreviations

NERICA: New rice for Africa
IBPGR: International board for plant genetic resources.
