# Supplementary material for: Conservation and utilization of African Oryza genetic resources
Source: Rice (N Y). 2013 Oct 29;6:29. doi: 10.1186/1939-8433-6-29 (PMC4883696; doi:10.1186/1939-8433-6-29)
Supplement: Supplementary file 1 — Additional file 1:Table S1. Germplasm collections held in various national genebanks. Table S2. Germplasm collected from selected countries and currently held in international genebanks. Table S3. Number of herbarium specimens of African Oryza species held in various herbaria globally. Table S4. Number of herbarium specimens of African Oryza collected from different African countries. (DOCX 30 KB) [file 12284_2013_79_MOESM1_ESM.docx]

**Additional files**

| **Country** | **Ex situ conservation facility** | **Species** | | | | | | | | |
| --- | --- | --- | --- | --- | --- | --- | --- | --- | --- | --- |
|  |  | ***O. barthii*** | ***O. longistaminata*** | ***O. punctata*** | ***O. eichingeri*** | ***O. brachyantha*** | ***O. glaberrima*** | ***O. sativa*** | ***Oryza sp.*** | ***Leersia sp.*** |
| Kenya | National Genebank of Kenya |  | 36 | 21 |  |  |  | 1004 | 6 | 3 |
| Uganda | Botany Department |  |  |  |  |  |  |  | 17 |  |
| Tanzania | National Plant Genetics Resources Centre | 2 |  | 3 |  |  |  | 218 | 35 |  |
| Zambia | Zambia Agricultural Research Institute |  |  |  |  |  |  | 202 |  |  |
| Zimbabwe | Genetic Resources and Biotechnology Institute | 1 | 1 | 1 |  |  |  |  | 55 | 5 |
| Benin | Centre de Recherches Agricoles Sud |  |  |  |  |  |  |  | 300 |  |
| Ethiopia | Genebank of the institute of biodiversity conservation |  |  |  |  |  |  | 15 | 118 |  |
| Malawi | Forest Research Institute of Malawi | 14 | 11 | 6 |  |  |  | 20 |  |  |
| Nigeria | National Centre for Genetic Resources and Biotechnology |  |  |  |  |  |  | 32 |  |  |
| Mozambique | National Center of Fotogenetic Resources |  | 26 | 1 |  |  |  | 344 | 4 | 5 |

**Additional file 1: Table S1: Germplasm collections held in various national genebanks**

Source: National information sharing mechanism and country reports on the state of plant genetic resources for food and agriculture

**Additional file 1: Table S2: Germplasm collected from selected countries and currently held in international genebanks**

| **Country** | ***O. barthii*** | ***O. longistaminata*** | ***O. punctata*** | ***O. eichingeri*** | ***O. brachyantha*** | ***O. glaberrima*** | ***O. sativa*** | ***Oryza sp.*** |
| --- | --- | --- | --- | --- | --- | --- | --- | --- |
| Kenya | - |  |  |  |  |  |  |  |
| Uganda |  |  | 4 | 18 |  |  |  |  |
| Tanzania | 9 | 20 | 23 | 1 | 2 | 6 | 322 | 4 |
| Zambia | 6 | 33 |  |  | 2 | 1 | 82 | 2 |
| Zimbabwe |  | 1 |  |  |  | 3 | 146 |  |
| Benin |  |  |  |  |  |  |  |  |
| Ethiopia |  | 11 |  |  |  |  | 10 | 1 |
| Malawi |  | 5 |  |  |  |  | 124 |  |
| Nigeria |  |  |  |  |  |  |  |  |
| Chad | 56 | 8 | 5 |  | 1 | 44 | 88 | 4 |
| Mozambique |  | 10 | 1 |  |  |  | 115 |  |
| Ghana |  | 2 | 5 | 2 |  | 102 | 326 | 1 |
| cote d'ivoire |  | 11 |  |  |  | 254 | 8880 | 1 |
| Niger | 10 | 6 | 3 |  | 2 | 20 | 14 | 8 |
| Nigeria | 12 | 20 | 10 |  |  | 1094 | 1768 | 8 |
| Cameroon | 29 | 18 | 9 |  | 5 | 94 | 161 | 3 |
| Sudan |  | 1 |  |  |  | 1 | 19 | 2 |
| Botswana | 1 | 6 |  |  |  |  | 15 |  |

Source: <http://www.genesys-pgr.org/>

**Additional file 1: Table S3: Number of herbarium specimens of African *Oryza* species held in various herbaria globally**

| **Institution** | ***O. barthii*** | ***O. longistaminata*** | ***O. punctata*** | ***O. eichingeri*** | ***O. brachyantha*** | ***O. glaberrima*** |
| --- | --- | --- | --- | --- | --- | --- |
| Royal Botanic Gardens, Kew | 7 | 28 | 4 | 4 | 2 | 2 |
| Missouri Botanical Garden | 3 | 11 | 6 | 7 |  | 5 |
| South African National Biodiversity Institute (SANBI) | 9 | 100 | 35 |  | 2 |  |
| MNHN - Museum national d'Histoire naturelle | 92 | 108 | 31 | 12 | 24 | 20 |
| Herbarium Senckenbergianum | 28 | 22 | 5 |  |  | 3 |
| University of Ghana – Ghana Herbarium | 6 | 36 | 8 |  |  | 1 |
| Netherlands Centre for Biodiversity Naturalis, | 27 | 24 | 5 | 2 | 10 | 4 |
| *Ecole de Faune de Garoua* | 9 | 17 |  |  |  |  |
| Herbarium of the University of Aarhus | 24 | 21 |  |  | 4 | 6 |
| Herbier du Bénin | 4 | 6 | 1 |  |  | 3 |
| Bioversity International- EURISCO | 5 |  |  |  | 1 | 4 |
| National Museum of Nature and Science, Japan | 2 |  |  | 2 | 1 | 1 |
| Australian National Herbarium |  |  |  | 6 |  |  |
| Botany (UPS) |  |  |  | 4 |  | 12 |
| Herbarium togoense |  | 1 | 1 |  |  | 2 |
| Real Jardin Botanico (Madrid), Vascular Plant Herbarium (MA) |  |  |  |  |  | 2 |

Source: **A**ccessed through GBIF data portal, Royal Botanic Gardens, Kew; South African National Biodiversity Institute (SANBI); MNHN - Museum national d'Histoire naturelle; Herbarium Senckenbergianum; University of Ghana – Ghana Herbarium; Netherlands Centre for Biodiversity Naturalis, section National Herbarium of the Netherlands; *Ecole de Faune de Garoua;* Herbarium of the University of Aarhus; Herbier du Bénin; Bioversity International- EURISCO; National Museum of Nature and Science, Japan; Australian National Herbarium Botany (UPS); Herbarium togoense; Real Jardin Botanico (Madrid), Vascular Plant Herbarium (MA), http://data.gbif.org/datasets/resource.

**Additional file 1: Table S4: Number of herbarium specimens of African *Oryza* collected from different African countries**

| **Country** | ***O. barthii*** | ***O. longistaminata*** | ***O. punctata*** | ***O. eichingeri*** | ***O. brachyantha*** | ***O. glaberrima*** |
| --- | --- | --- | --- | --- | --- | --- |
| Kenya | 0 | 1 | 2 | 1 |  |  |
| Uganda | 0 | 1 | 2 | 1 |  |  |
| Tanzania | 0 | 6 | 15 | 5 |  |  |
| Zambia | 1 | 8 |  |  | 2 |  |
| Zimbabwe | 1 | 6 | 3 |  |  |  |
| Benin | 21 | 21 | 1 |  |  | 3 |
| Ethiopia | 0 | 1 |  |  |  |  |
| Malawi | 0 | 2 | 1 |  |  |  |
| Nigeria | 7 | 5 | 2 |  |  |  |
| Chad | 2 | 1 |  |  |  |  |
| Mozambique | 0 | 3 | 1 |  |  |  |
| Ghana | 5 | 32 | 9 |  |  | 1 |
| cote d'ivoire | 4 | 3 | 1 | 1 | 6 | 1 |
| Niger | 0 | 0 |  |  |  |  |
| Cameroon | 13 | 24 | 6 |  |  | 6 |
| Sudan | 5 |  | 5 |  |  | 1 |
| Botswana | 4 | 25 |  |  |  |  |
| South Africa | 0 | 31 | 8 |  |  |  |
| Guinea |  |  |  |  | 1 |  |
| Mali | 7 | 9 |  |  | 1 | 7 |
| Sierra Leone |  | 2 |  |  | 1 |  |
| Senegal | 20 | 13 |  |  | 4 | 1 |
| Madagascar |  | 5 | 1 |  |  |  |
| Namibia |  | 12 | 1 |  |  |  |
| Somalia |  |  | 2 |  |  |  |
| Swaziland |  |  | 6 |  |  |  |
| Togo |  | 1 | 1 |  |  | 2 |
| Burkina Faso | 24 | 27 |  |  |  | 10 |
| Angola | 2 | 1 |  |  |  |  |
| Liberia |  |  |  |  |  | 4 |
| Central African republic | 4 |  |  |  |  |  |
| Country not specified | 96 | 110 | 31 | 14 | 26 | 29 |

Source: <http://www.gbif.org/>
