# Supplementary figures and images for: Conservation and utilization of African Oryza genetic resources
Source: Rice (N Y). 2013 Oct 29;6:29. doi: 10.1186/1939-8433-6-29 (PMC4883696; doi:10.1186/1939-8433-6-29)

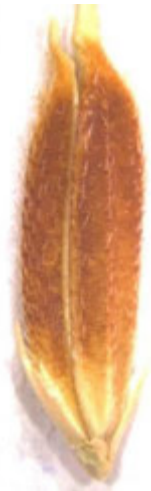

a

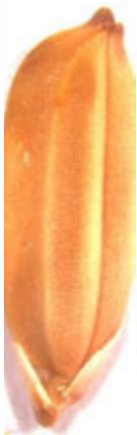

b

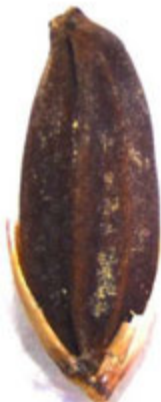

c

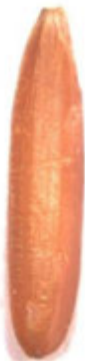

d

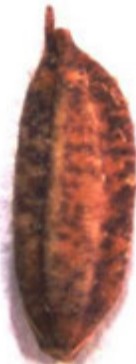

e

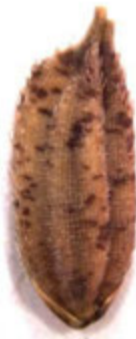

f

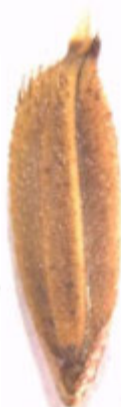

g

2mm

Supplement: Supplementary file 2 — Authors’ original file for figure 1 [file 12284_2013_79_MOESM2_ESM.pdf]

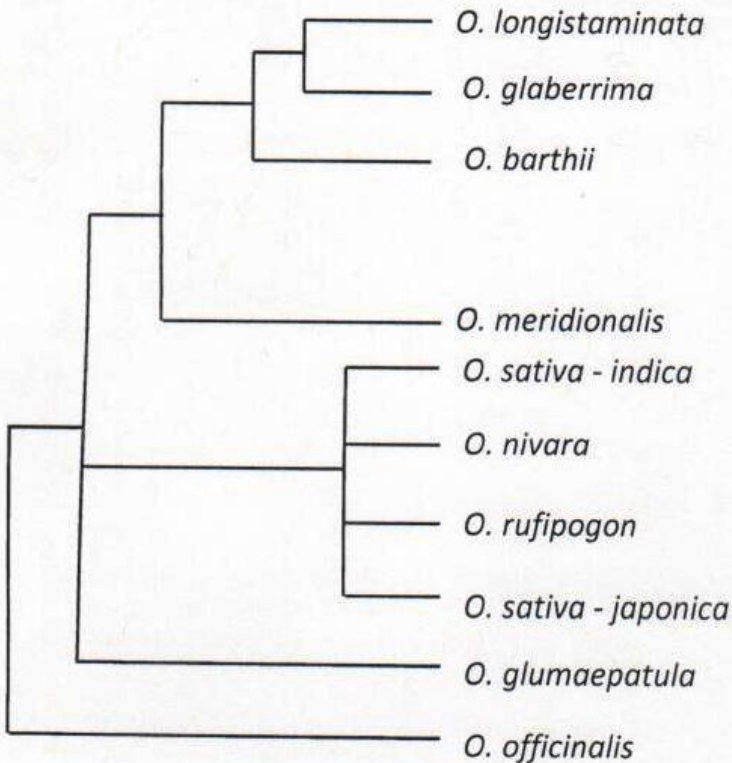

Supplement: Supplementary file 3 — Authors’ original file for figure 2 [file 12284_2013_79_MOESM3_ESM.pdf]

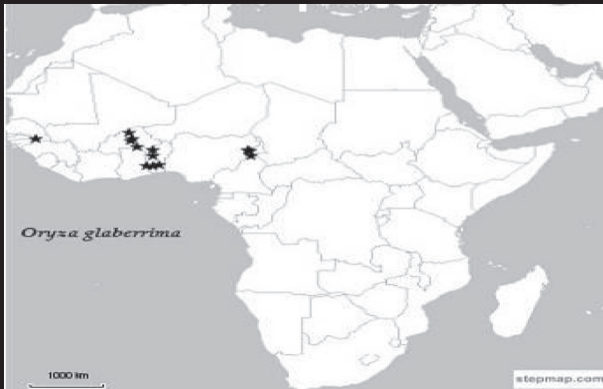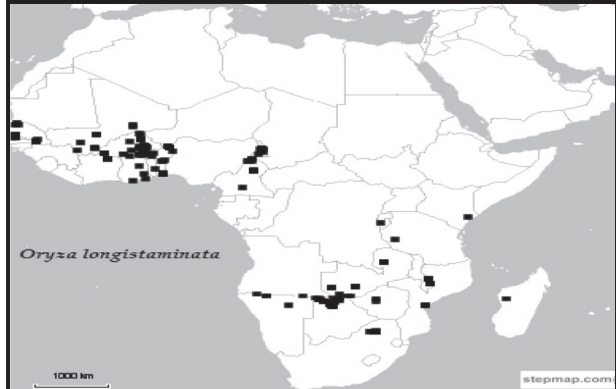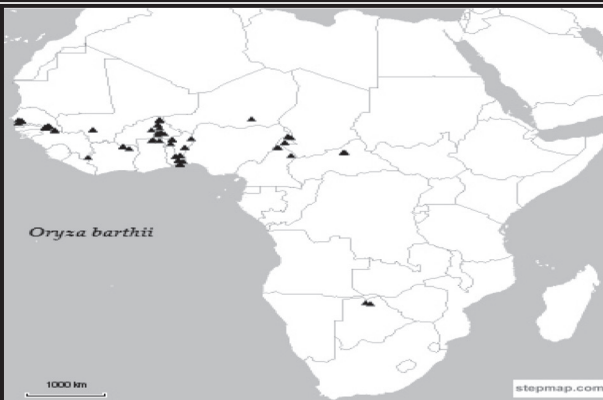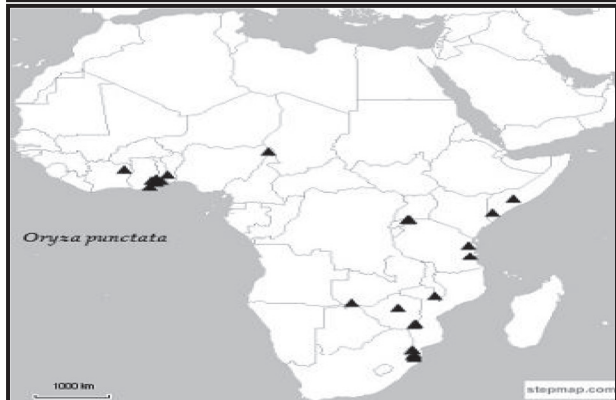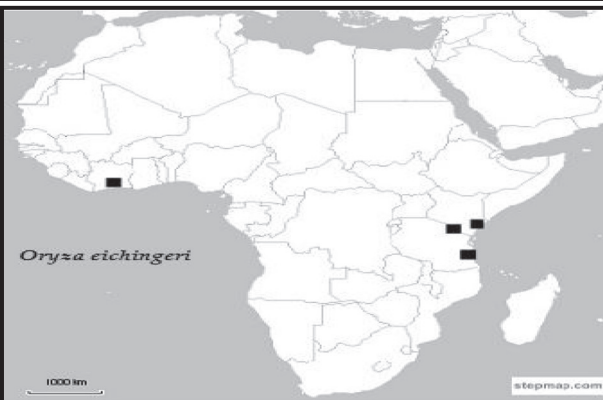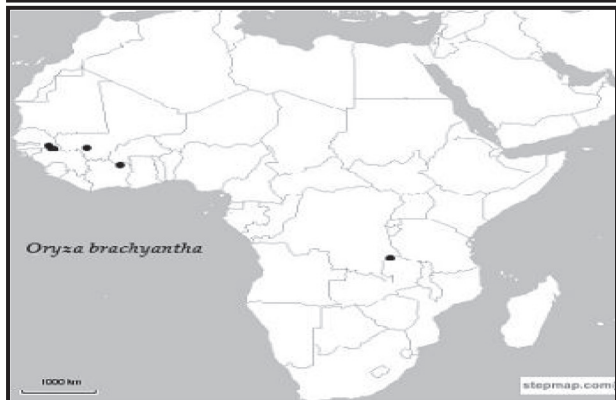

Supplement: Supplementary file 4 — Authors’ original file for figure 3 [file 12284_2013_79_MOESM4_ESM.pdf]

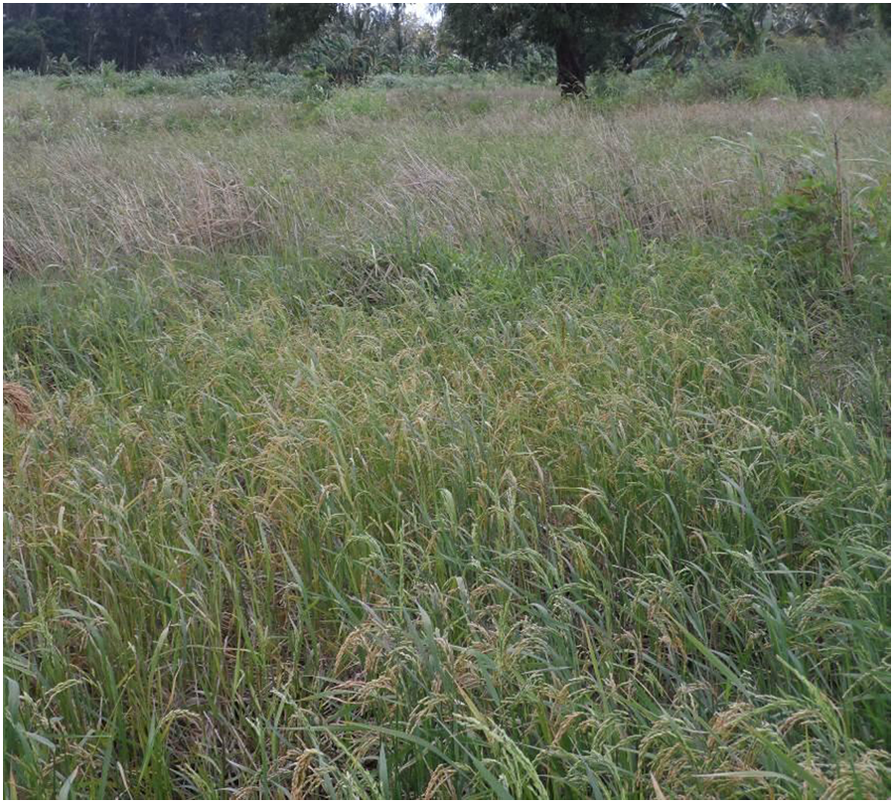

Supplement: Supplementary file 5 — Authors’ original file for figure 4 [file 12284_2013_79_MOESM5_ESM.tiff]

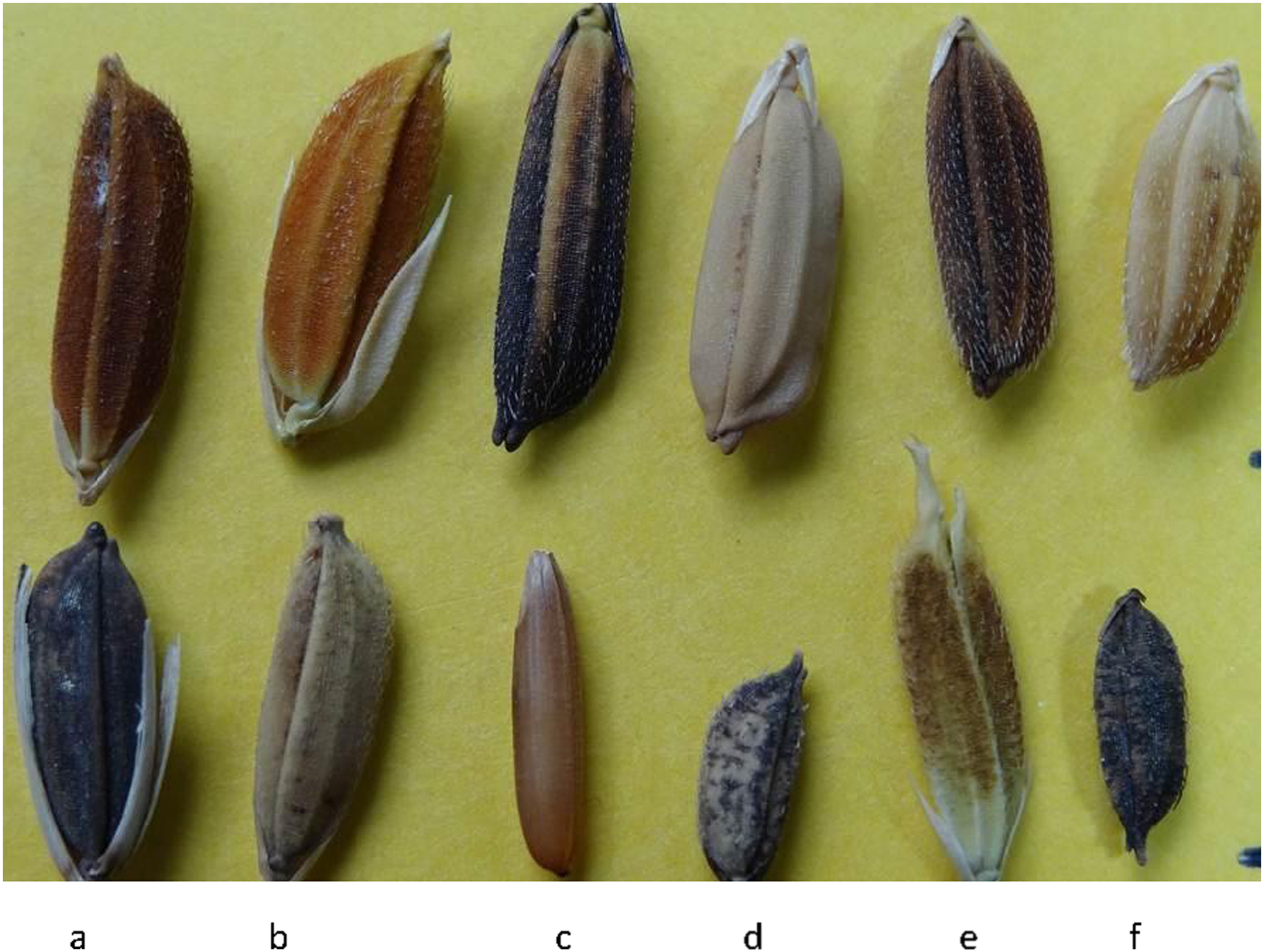

Supplement: Supplementary file 6 — Authors’ original file for figure 5 [file 12284_2013_79_MOESM6_ESM.tiff]

g

f

e

d

c

b

a


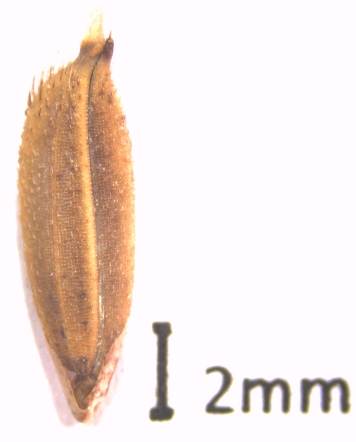

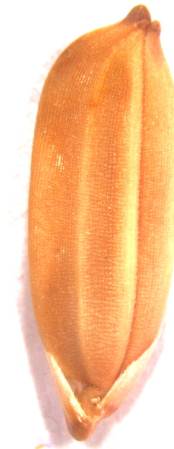

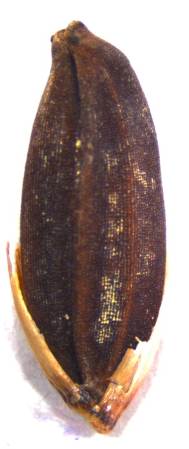

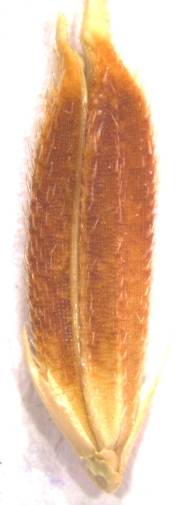

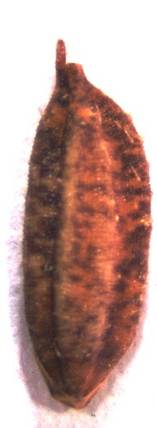

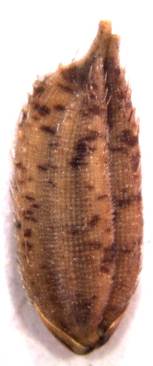

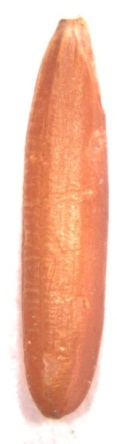


Figure 1

Supplement: Supplementary file 7 — Authors’ original file for figure 6 [file 12284_2013_79_MOESM7_ESM.doc]
